# Dispensing and Dr. Addison

**Published:** 1920-01-24

**Authors:** 


### Dispensing and Dr. Addison.

### PANEL CHEMISTS' DECISION.

A Conference of Chemists' Associations delegates has been held in London to consider the terms of service offered for 1920 in the dispensing of medicines under the National Insurance Act. There was a large attendance, and it was eventually decided to send a deputation to Dr. Addison to place before him the chemists' views regarding remuneration for dispensing medicines.

It is understood that 50 per cent. extra on the fees was requested, but a compromise was arrived at which the conference decided to accept, and delegates go back to their associations recommending them to accept service for 1920; a spirit which might with advantage be displayed by other sections of the community.

A new regulation now requires each district to make adequate arrangements for supplying Insurance medicines after usual shop hours, and it is probable that a "rota" will be established by groups of chemists in the different areas and that each will give late attendance for a specified number of nights every month. It is proposed that insured persons will be informed, by notices in the chemists' shop-windows, as to where they may have Insurance prescriptions dispensed after the usual hours.

